# Rhus Rad. *vs.* Rhus Tox.

**Published:** 1888-06

**Authors:** Robert Farley

**Affiliations:** Phœnixville, Pa.


### RHUS RAD. VERSUS RHUS TOX.

PHŒNIXVILLE, PA., May 3d, 1888.

EDITORS HOMŒOPATHIC PHYSICIAN: In reading the discussion of Rhus rad. and Rhus tox. by the members of the "Syracuse Hahnemannian Club" I find cause for dissent. Dr. Seward unequivocally declares nature to be unable to correct or eradicate the symptoms due to Rhus rad. poisoning. This is certainly a mistake, as I know from observation and much painful personal experience.

I do not know of any case that will illustrate the matter better than my own. I am extremely susceptible to the action of Rhus rad., the wind blowing over the growing plant in the early morning being sufficient to cause me intolerable suffering. When a boy I was often exposed and suffered accordingly, and received the crudest kind of local treatment, and yet always convalesced, with crop of furuncles, and I could cite scores of other instances of perfect recovery without any treatment.

And now to dissent again. Dr. Hooker says the poison is carried by the hands "to the face and genitals." This is another mistake which painful personal experience enables me to correct. Once the drug has affected the system, it then shows its affinity for the face, especially the orbitial region and the sexual organs, in spite of the greatest care to avoid spreading it by contact with the hands. I have been blind for days and my penis and scrotum have been swollen as large as my head and the color of a boiled lobster, and all this after carefully washing my hands in ammonia water and absolutely not allowing my hands to come in contact with my face or sexual organs until after symptoms had manifested themselves there. This way of studying Rhus rad. has made an indelible impression on my memory.

ROBERT FARLEY, M. D.

